# Construction of Nomogram-Based Prediction Model for Clinical Prognosis of Patients with Stage II and III Colon Cancer Who Underwent Xelox Chemotherapy after Laparoscopic Radical Resection

**DOI:** 10.1155/2022/7742035

**Published:** 2022-09-30

**Authors:** Qiang Sun, Kai Xu, Shifeng Teng, Wenqiang Wang, Wei Zhang, Xinxing Li, Zhiqian Hu

**Affiliations:** Department of General Surgery, Tongji Hospital, School of Medicine, Tongji University, Shanghai 200092, China

## Abstract

**Objective:**

To construct a nomogram-based prediction model for the clinical prognosis of patients with stage II and III colon cancer who underwent Xelox chemotherapy after laparoscopic radical resection based on large data sets.

**Methods:**

A total of 7,832 patients with colorectal cancer who received postoperative Xelox-based chemotherapy were screened from the Surveillance, Epidemiology, and End Results database (USA) as the training data set. In addition, 348 domestic patients were screened as the validation data set. Multivariate Cox regression analysis was performed to identify variables for inclusion in the nomogram-based prediction model. The predictive accuracy of the model was assessed using C-index and calibration curve.

**Results:**

Age, cell differentiation, nerve invasion, T and N stages of tumours, number of dissected lymph nodes, and carcinoembryonic antigen (CEA) level were found to influence the efficacy of postoperative chemotherapy. The nomogram-based prediction model was successfully constructed. The C-index of both the training set and validation set were higher than those of the 7th edition of TNM staging system published by the American Joint Commission on Cancer (C − index of training data set = 0.728, C − index of validation data set = 0.734). The prediction results of the model in the calibration curve showed a good fit with the actual situation.

**Conclusion:**

We successfully constructed a nomogram-based model to predict the clinical prognosis of patients with colorectal cancer receiving postoperative Xelox-based chemotherapy after laparoscopic radical resection, which showed good clinical application value for predicting the efficacy of postoperative Xelox-based chemotherapy in patients with colorectal cancer.

## 1. Introduction

Colorectal cancer is a common malignant tumour in China and is associated with a high mortality rate. Currently, surgical resection supplemented with chemotherapy is the main treatment modality for colorectal cancer. Most patients undergoing surgery have advanced stage disease and are at a high risk of postoperative recurrence and/or metastasis. Therefore, chemotherapy is typically used to achieve disease control in clinical settings [1]. The National Comprehensive Cancer Network guidelines recommend Xelox-based chemotherapy (Oxaliplatin: 130 mg/m^2^, intravenically given, 2 h, d1; Capecitabine: 1800 mg/(m^2^·d), two oral cycles, d1-14, every 21 days) as the first-line regimen after surgery for colorectal cancer. It is a widely used chemotherapy regimen in clinical settings owing to the ease of administration and high efficacy [2]. Despite the advances in surgery and chemotherapy regimens, a large proportion of patients with colorectal cancer develop postoperative recurrence and metastasis, leading to poor prognosis. Thus, identification of prognostically relevant clinical factors and their use to predict the treatment outcomes may help individualise the treatment plan and improve the prognosis of patients [3]. Nomograms assign scores for various influencing factors calculated by the statistical model; the obtained total score of individual risk can help predict the risk of morbidity. Therefore, in this study, a nomogram-based model was constructed to predict the prognosis of patients with colorectal cancer receiving postoperative Xelox-based chemotherapy by analysing the relevant data.

## 2. Subjects and Methods

### 2.1. Subjects

Data pertaining to patients with colorectal cancer recorded in the Surveillance, Epidemiology, and End Results (SEER) database (USA) from 2011 to 2016 was used as the training data set. The inclusion criteria were as follows: age ≥ 18 years; primary tumour, located at the colorectum (code: C18.0, C18.2–C18.7, C19.0, C20.0, C20.X01); pathological diagnosis: adenocarcinoma (code: M81400); patients who underwent surgery (code: 20~80) and received postoperative Xelox-based chemotherapy. The exclusion criteria were as follows: patients with incomplete clinically relevant data, including age, gender, tumour stage and grade, laboratory examination results, and follow-up data. According to the inclusion and exclusion criteria, 7,832 patients were finally screened as the training data set. Simultaneously, a validation data set was established. From 2014 to 2016, a total of 348 patients who underwent colorectal resection and Xelox-based chemotherapy were identified from the electronic medical record system at our hospital. Complete clinical information was available for all patients. Identical inclusion and exclusion criteria were adopted for both the training and validation data sets.

### 2.2. Methods

#### 2.2.1. Data Collection

Detailed clinical data were retrieved for patients in both the training and validation data sets, including gender, age, tumour location, tumour stage, cell differentiation, depth of cancer invasion, lymph node metastasis, and carcinoembryonic antigen (CEA) level.

#### 2.2.2. Follow-Up

Complete follow-up data was available for all patients in the training data set. All patients in the validation set were followed up for 3 years; the patients were followed up once a month in the first year, every three months in the second year, and at six-month intervals in the third year. Follow-up data of patients were obtained mainly through face-to-face interview in the doctors' office or through telephonic contact. If the patient could not be contacted, the relevant information was obtained from the patient's family or community doctors. A follow-up record was established for every patient to document the detailed prognosis of patients after discharge. According to the follow-up results, the overall survival (OS) and progression-free survival (PFS) were calculated and a detailed list was made, which were used as the end points of the study. OS was defined as the time from diagnosis to death or the end of follow-up; PFS was defined as the time from diagnosis to the first tumour progression, death, or the end of follow-up.

#### 2.2.3. Statistical Analysis

Data were sorted and analysed using SPSS Statistics 26 (IBM) and R language 3.6.2 (Bell Laboratories). The categorical variables were expressed as percentage (%) and between-group differences assessed using the chi-squared test. For the analysis of prognostic factors, univariate analysis was performed with the log-rank *χ*^2^ test. Variables that showed a significant association with prognosis on univariate analysis (*P* < 0.05) were included in multivariate Cox regression analysis to identify the factors influencing OS and PFS. Finally, the nomogram-based prediction model was constructed using variables screened by the multivariate analysis. The accuracy of the model was verified by Harrell's C-statistic and calibration curve. Two-tailed *P* < 0.05 were considered indicative of statistical significance. Calibration, which refers to how closely the predicted probabilities by the nomogram agree with the observed survival probabilities, was visually assessed by plotting actual survival probabilities against predicted survival probabilities for each group. The horizontal and vertical axes of the calibration plot showing the predicted versus the observed probability of the 5-year overall survival and progression-free survival. The gray line represents the optimal line in case of complete concordance between predicted and observed progression-free survival. Decision curve analysis (DCA) was used to evaluate the clinical benefits and utility of the nomogram compared with an American Joint Council on Cancer (AJCC) staging system alone.

## 3. Results

### 3.1. Baseline Data of Patients

The 7,832 patients in the training data set included 3,822 males and 4,010 females (mean age: 54.7 ± 8.9 years); the OS was 30.4 (10.3–36) months and PFS was 18.3 (6.2–31.8) months. The 348 patients included in the validation data set included 172 males and 176 females (mean age: 53.2 ± 8.5 years; the OS was 29.8 (10.1–36) months and PFS was 18.4 (5.9–32.7) months). The baseline data in the two data sets are compared in Table 1.

### 3.2. Factors Influencing the Efficacy of Postoperative Chemotherapy

On univariate analysis, age, cell differentiation, nerve invasion, T and N stages of tumours, number of dissected lymph nodes, and CEA level were found to have a significant influence on OS and PFS (*P* < 0.05). Multivariate Cox regression analysis showed that the above variables were independent predictors of OS and PFS (*P* < 0.05) (Table 2).

### 3.3. Construction and Validation of Nomogram-Based Prediction Model

**Figure 1:**
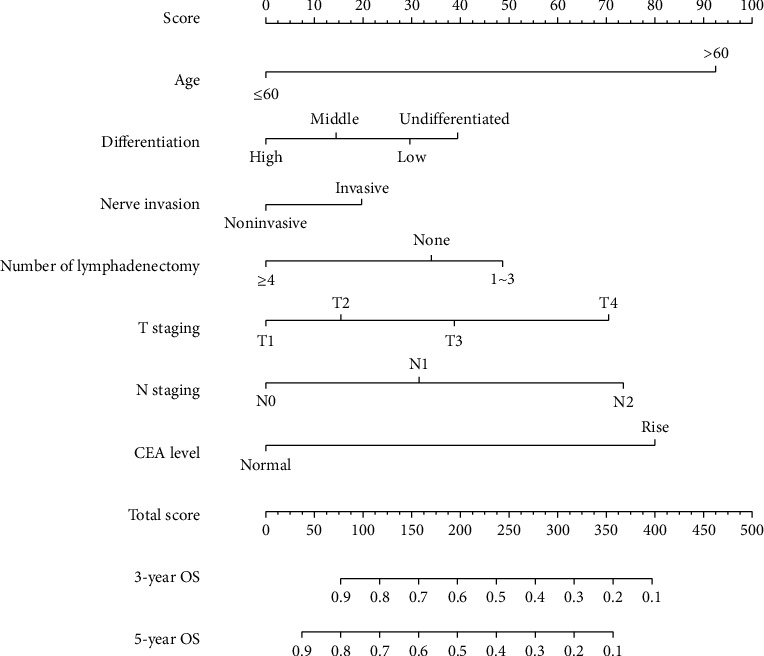
OS nomogram of 3-year and 5-year prognoses for colorectal cancer patients.

**Figure 2:**
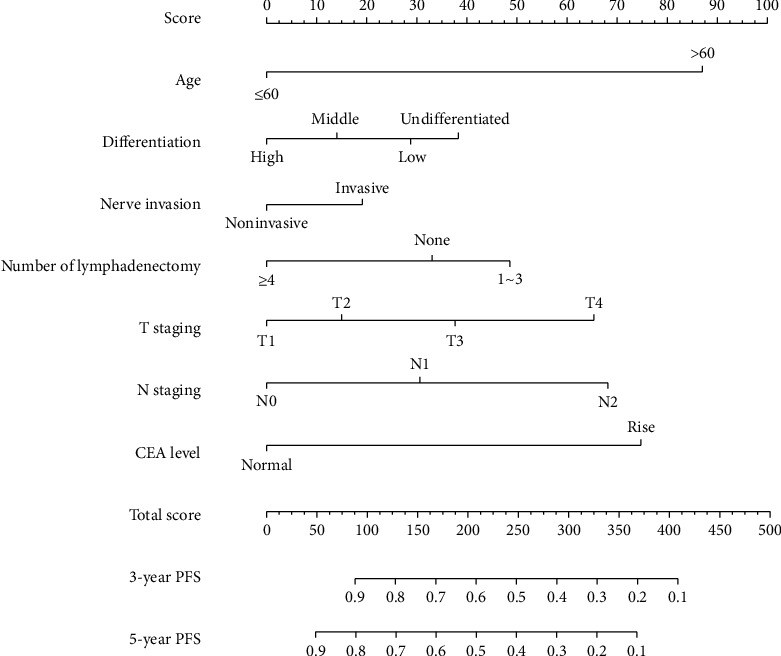
PFS nomogram of 3-year and 5-year prognoses for colorectal cancer patients.

Cox regression analysis identified seven variables that influenced the prognosis of patients with colorectal cancer receiving Xelox-based chemotherapy. The nomogram-based prediction model was constructed; on the basis of the model, individualised risk scoring was performed, and the 3-year and 5-year survival rates (OS and PFS) were predicted (Figures 1 and 2).

**Figure 3:**
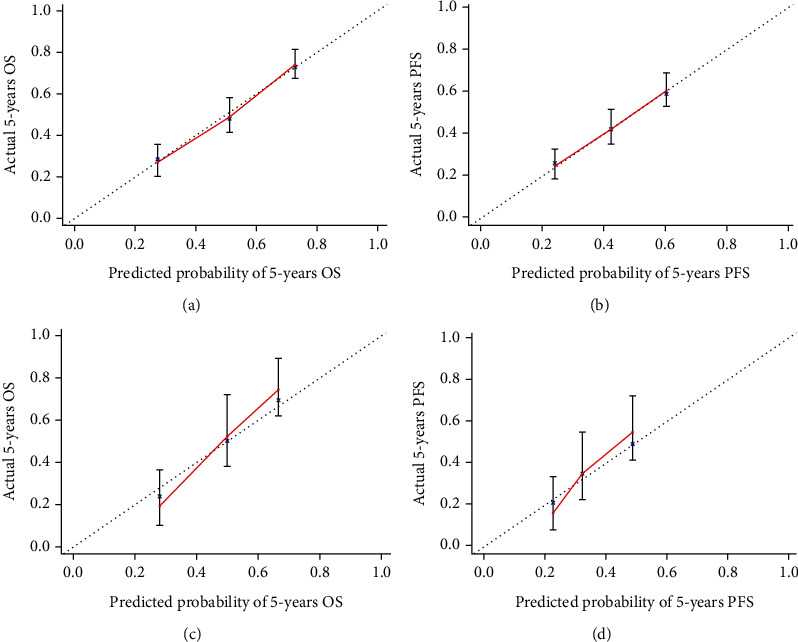
Fitting curve (OS and PFS) between prediction model and actual survival of patients: (a, b) training data set; (c, d) validation data set.

**Figure 4:**
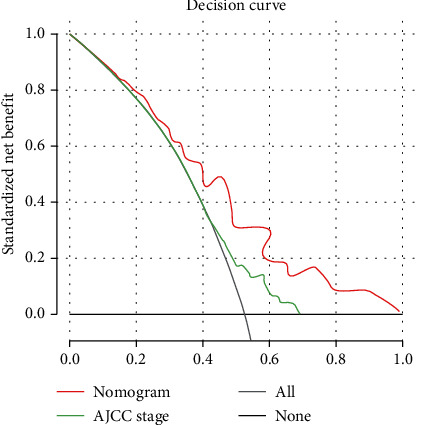
Decision curve analysis for the nomogram and the AJCC stage.

For OS, the C-index of the training data set and validation data set was 0.792 and 0.753, respectively. For PFS, the C-index was 0.783 and 0.761, respectively. All these values were higher than those of the 7th edition of TNM staging system published by the American Joint Commission on Cancer (AJCC) (C − index of training data set = 0.728, C − index of validation data set = 0.734). The results suggested a slightly more accurate prediction ability of the model compared with the traditional staging method. In addition, the calibration curve was drawn using the survival rate predicted by the model as the horizontal ordinate and the actual survival as the longitudinal ordinate. For the end-point indicators OS and PFS, the results of the prediction model showed a good fit with the actual situation; this suggested high discriminative ability and accuracy of the prediction model constructed in this study (Figure 3). The 5-year DCA curves also revealed that the nomogram had better clinical performance than the AJCC staging system among all study subjects (Figure 4).

## 4. Discussion

### 4.1. Application of Xelox Regimen in Patients with Colorectal Cancer after Surgery

Currently, colorectal cancer is one of the common malignant tumours of the digestive tract and is associated with high mortality and poor prognosis. Surgery is the only potential curative treatment recognised in clinical practice [1]. However, owing to the lack of obvious symptoms in the early stage of the disease, patients with colorectal cancer are typically diagnosed in the middle and late stages; most of these patients are past the optimal time to achieve radical cure. Moreover, there is a high risk of postsurgical recurrence and metastasis [4]. Therefore, postoperative chemotherapy is typically administered to patients with colorectal cancer who undergo surgery. However, patients with colorectal cancer often have digestive dysfunction, physical weakness, and multiple comorbid conditions. All these factors contribute to chemotherapy intolerance; therefore, selection of the appropriate chemotherapy regimen is a key imperative for these patients.

Xelox-based chemotherapy (also known as the CapeOX regimen) consists of oxaliplatin injection administered in combination with oral Xeloda. Owing to its efficacy and ease of administration, it is used as the main postoperative adjuvant chemotherapy regimen for patients with colorectal cancer in clinical settings [5]. However, approximately 50% of patients who received postoperative Xelox-based chemotherapy were found to develop recurrence and metastasis at different time points after surgery; in addition, the prognosis of these patients is still not very ideal [6]. Therefore, construction of statistical models based on appropriate clinical indicators to predict the prognosis of patients can facilitate individualised treatment decision-making and help improve the prognosis of patients.

### 4.2. Factors Influencing the Efficacy of Postoperative Chemotherapy in Patients with Colorectal Cancer

In this study, age, cell differentiation, nerve invasion, T and N stages of tumours, number of dissected lymph nodes, and CEA level were found to influence OS and PFS. Our results are consistent with those of previous studies, but not exactly the same.

In our study, age was the most important determinant of prognosis. The older the patient, the worse was the prognosis. Therefore, the benefit of surgical treatment for older patients should be carefully considered based on individualised analysis and assessment of the general condition of the patient [7]. For elderly patients with poor tolerance, the risk of surgery may outweigh the benefits. Additionally, dissection of 1–3 lymph nodes was found to be more dangerous than no dissection; therefore, clinicians should consider increasing the number of dissected lymph nodes in patients scheduled to undergo lymph node dissection [7, 8]. The prognostic value of cell differentiation, nerve invasion, and tumour stage was in line with that found in previous studies [9–11]. In this study, levels of CEA were included in the model as factors influencing the prognosis. The final results showed that all three factors were independent predictors of prognosis. As a proteoglycan compound of the digestive system, CEA is a commonly used tumour marker; the correlation of the CEA level with the prognosis of patients with colorectal cancer is well documented [12–15].

### 4.3. Advantages of the Prediction Model Constructed in This Study

Nomogram-based prediction models provide visual representation of individual risk assessment. It employs multiple clinical indicators, scores the value of each indicator, and finally predicts the corresponding situation of patients according to the total score of individuals. Use of nomograms to predict the incidence and prognosis is a current research hot spot. It can intuitively and accurately display complex mathematical formulas in the form of images and has high clinical application value [16]. However, the prediction model for the efficacy of postoperative chemotherapy in patients with colorectal cancer has rarely been reported.

In this study, we constructed a nomogram-based prediction model using variables identified on multivariate analysis; the prediction model was found to accurately predict individual prognosis. The model showed high discriminative ability and accuracy in the validation cohort. In addition, we compared our nomogram-based model with the 7th edition of the TNM staging system published by the AJCC; our model showed higher prediction ability in both the training and validation data sets. Visual analysis of the calibration curve showed a good fit of the prediction of the training data set with the actual situation; however, the fit of the verification data set showed a certain deviation. This deviation may be attributable to bias caused by insufficient sample size, ethnic differences, and variable selection of the verification data set. The DCA results also demonstrated that our nomogram provided greater clinical value than the AJCC grading system.

### 4.4. Limitations and Reflection

The prediction model constructed in this study effectively predicted the efficacy of postoperative chemotherapy in patients with colorectal cancer; however, some limitations of the study should be considered while interpreting the results. Firstly, due to the limitations of SEER data, the grouping criteria for some indicators were different from those used in actual clinical practice. For example, for the grouping of the number of dissected lymph nodes, a cut-off value of 12 lymph nodes is used in clinical settings; however, four lymph nodes were used as the cut-off value in the database [17]. Secondly, there were inevitable limitations during data acquisition owing to the retrospective nature of the study. Moreover, there may be a certain bias in the selection of variables. Further studies are required to confirm our results and to further improve the prediction model.

## 5. Conclusion

Based on the SEER database and our institutional medical record database, we successfully constructed a prediction model for OS and PFS. The model showed good clinical application value for predicting the efficacy of postoperative Xelox-based chemotherapy in patients with colorectal cancer. Both the training and validation data sets showed higher predictive ability when compared with the 7th edition of the TNM staging system published by the AJCC.

## Figures and Tables

**Table 1:** Comparison of baseline data of patients.

Variable	Training data set (*n* = 7832) *n* (%)	Validation data set (*n* = 348) *n* (%)	*χ^2^*	*P*
Gender			0.052	0.819
Male	3822 (48.8)	172 (49.4)		
Female	4010 (51.2)	176 (50.6)		
Age			0.167	0.682
≤60	3305 (42.2)	143 (41.1)		
>60	4527 (57.8)	205 (58.9)		
Tumour location			0.146	0.703
Rectum	5325 (68.0)	240 (69.0)		
Colon	2507 (32.0)	108 (31.0)		
Cell differentiation			0.496	0.920
High	407 (5.2)	16 (4.6)		
Middle	5864 (74.9)	266 (76.4)		
Low	1253 (16.0)	53 (15.2)		
Undifferentiated	308 (3.9)	13 (3.7)		
Nerve invasion			0.001	0.976
Invasive	6657 (85.0)	296 (85.1)		
Noninvasive	1175 (15.0)	52 (14.9)		
T staging			0.407	0.939
T1	289 (3.7)	12 (3.4)		
T2	971 (12.4)	46 (13.2)		
T3	4825 (61.6)	216 (62.1)		
T4	1747 (22.3)	74 (21.3)		
Number of lymphadenectomy			0.250	0.883
None	110 (1.4)	6 (1.7)		
1~3	71 (0.9)	3 (0.9)		
≥4	7651 (97.7)	339 (97.4)		
N staging			5.009	0.082
N0	4104 (52.4)	163 (46.8)		
N1	2318 (29.6)	109 (31.3)		
N2	1410 (18.0)	76 (21.8)		
CEA level			0.618	0.432
Rise	3344 (42.7)	156 (44.8)		
Normal	4488 (57.3)	192 (55.2)		

**Table 2:** Analysis of influencing factors of OS and PFS in colorectal cancer patients during training data set.

Variable	OS				PFS			
	Single-factor analysis		Multifactor analysis		Single-factor analysis		Multifactor analysis	
	OR (95% CI)	*P*	OR (95% CI)	*P*	OR (95% CI)	*P*	OR (95% CI)	*P*
Gender	—	0.137	—	—	—	0.231	—	—
Age								
≤60	1.00	—	—	—	1.00	—	—	—
>60	2.17 (1.97~2.64)	0.002	2.56 (1.98~2.79)	<0.001	2.31 (1.86~2.53)	0.003	2.43 (1.91~2.68)	<0.001
Tumour location	—	0.261	—	—	—	0.164	—	—
Differentiation								
High	1.00	—	—	—	1.00	—	—	—
Middle	1.34 (1.13~1.56)	0.005	1.11 (1.03~1.23)	<0.001	1.23 (1.13~1.54)	<0.001	1.14 (1.03~1.21)	<0.001
Low	1.53 (1.38~1.87)	<0.001	1.25 (1.12~1.37)	0.004	1.47 (1.31~1.81)	<0.001	1.28 (1.18~1.33)	0.001
Undifferentiated	2.16 (1.76~2.68)	<0.001	1.36 (1.26~1.48)	<0.001	2.23 (1.85~2.45)	0.017	1.34 (1.21~1.48)	0.021
Nerve invasion								
Invasive	1.00	—	—	—	1.00	—	—	—
Noninvasive	2.11 (1.82~2.41)	0.014	1.37 (1.21~1.51)	<0.001	2.25 (1.91~2.42)	0.001	1.41 (1.24~1.67)	<0.001
T staging								
T1	1.00	—	—	—	1.00	—	—	—
T2	1.23 (1.14~1.42)	<0.001	1.13 (1.04~1.25)	<0.001	1.22 (1.11~1.55)	<0.001	1.15 (1.04~1.26)	<0.001
T3	1.42 (1.31~1.63)	0.002	1.31 (1.21~1.54)	<0.001	1.67 (1.42~1.89)	<0.001	1.41 (1.28~1.61)	<0.001
T4	2.43 (2.01~2.83)	<0.001	1.54 (1.36~1.78)	0.006	2.43 (1.93~2.75)	0.004	1.57 (1.35~1.81)	0.017
Number of lymphadenectomy								
None	1.23 (0.92~2.12)	0.164	1.42 (0.97~2.18)	0.761	1.23 (0.93~2.01)	0.182	1.51 (0.94~2.12)	0.687
1~3	1.51 (1.02~2.24)	0.031	1.83 (1.15~2.63)	0.003	1.41 (1.12~2.31)	0.042	1.91 (1.32~2.28)	0.003
≥4	1.00	—	—	—	1.00	—	—	—
N staging								
N0	1.00	—	—	—	1.00	—	—	—
N1	1.91 (1.62~2.15)	<0.001	1.61 (1.37~1.83)	0.006	1.82 (1.59~2.03)	<0.001	1.72 (1.47~1.93)	0.002
N2								
CEA level								
Normal	1.00	—	—	—	1.00	—	—	—
Rise	2.57 (2.28~2.87)	<0.001	1.98 (1.69~2.18)	0.021	2.47 (2.18~2.73)	0.029	2.01 (1.83~2.34)	0.017

## Data Availability

The experimental data used to support the findings of this study are available from the corresponding authors upon request.
